# Assessing knowledge about acupuncture: A survey of people with back pain in the UK

**DOI:** 10.1016/j.ctim.2016.10.005

**Published:** 2016-12

**Authors:** Maddy Greville-Harris, John Hughes, George Lewith, Christina Liossi, Peter White, Cynthia A. Graham, Felicity L. Bishop

**Affiliations:** a Department of Psychology, University of Southampton, Southampton, UK; b Royal London Hospital for Integrated Medicine, UCLH NHS Trust, London, UK; c School of Medicine, University of Southampton, Southampton, UK; d Faculty of Health Sciences, University of Southampton, Southampton, UK

**Keywords:** Acupuncture, Health knowledge, Attitudes, Practice, Statutory regulation, Survey, Back pain

## Abstract

**Objectives:**

Despite the prevalence of acupuncture treatment in the UK, and the increasing evidence of safety and effectiveness, the information presented to patients by practitioners frequently contains inaccuracies. As knowledge of treatment affects both patient decision-making and treatment outcomes, this study aimed to establish what is known about acupuncture in a sample of people who had, and had not, previously experienced acupuncture.

**Design:**

A 15-item questionnaire was constructed to assess knowledge of acupuncture.

**Setting:**

Online survey of people with a history of back pain.

**Results:**

202 participants completed the questionnaire. 66.8% of the sample was female and 33.2% male, with a mean age of 35 years (range 18–74 years). 87.6% had back pain in the past six months, 44.1% currently. 21.8% had previously received acupuncture, and 69.8% had previously read or heard information about acupuncture. On average participants answered 11.03 of 15 questions about acupuncture correctly (SD = 2.64). Items relating to common concerns about acupuncture, acupuncture efficacy, and types of acupuncture were correctly answered by ≥80% of participants. Participants possessed less knowledge of accessibility, Government legislation, and methods of administration.

**Conclusions:**

The study identified key gaps in knowledge about acupuncture among patients. In particular, many participants were unaware that acupuncture is available from the UK National Health Service and that acupuncturists are not subject to statutory regulation in the UK. These knowledge gaps should be addressed in order to increase people’s understanding of and access to acupuncture.

## Highlights

• 202 adults with back pain completed a survey assessing knowledge of acupuncture.
• More than 80% correctly answered questions on efficacy, side-effects, and styles.
• Fewer people knew about public sector provision, regulation, or electro-acupuncture.
• Addressing these knowledge gaps would improve public awareness of acupuncture.

## 1 Introduction

Acupuncture is arguably the most researched and widely accepted complementary medicine modality. Acupuncture is extensively practiced in the UK, with an estimated 3.8 million acupuncture treatments delivered in 2009.^1^ A recent meta-analysis based on individual patient data from nearly 18,000 patients demonstrated acupuncture is effective for a range of painful conditions.^2^ UK National Institute for Health and Care Excellence (NICE) clinical guidelines currently recommend acupuncture for low back pain, tension-type headache, and migraine.^3^ Acupuncture is also a relatively safe form of treatment, with a large prospective observational study of 229,230 patients finding incidence of serious adverse effects from acupuncture to be low.^4^

Despite the prevalence of acupuncture treatment in the UK, and the increasing evidence of safety and effectiveness, the information presented to patients by practitioners may contain inaccuracies. Bishop and Salmon^5^ analysed 401 information leaflets from practising UK acupuncturists and found inconsistencies and some inaccuracies. Claims about the efficacy of acupuncture for different health conditions were not always supported by empirical evidence, leaflets did not always refer to professional bodies, and some did not present information about risks or safety.^5^ Additional research has found UK patients who consent to receive acupuncture treatment do not always have an accurate or complete understanding of the treatment, believing in common misconceptions, such as linking acupuncture to ‘voodoo’ or ‘witchcraft’, and describing concerns about treatment, particularly the application of acupuncture needles.^6^, ^7^

Accurate knowledge about treatment during the decision-making process is important. Making treatment choices that are in line with one’s attitudes towards treatment and based on accurate knowledge (i.e. an ‘informed choice’), is in line with health policy in the UK.^8^ The Power of Information Report issued by the Department of Health (2012) recommended that patients have reliable, relevant and easily accessible information to support them in making treatment decisions.^9^ Thus, it is recognised that accurate knowledge is vital for making informed decisions about treatment options.

As well as being integral to treatment decision-making, accurate knowledge of an intervention is also a significant factor in determining treatment outcome, as patients’ beliefs about acupuncture have been shown to predict treatment outcomes.^10^, ^11^ Kalauokalani et al.^12^ carried out a randomized clinical trial of massage and acupuncture for back pain in which patients’ expectations of massage and acupuncture were assessed before they were randomized to receive one of these treatments. High expectation of a specific treatment’s success (i.e. of acupuncture’s efficacy for those who received acupuncture) was associated with positive treatment outcomes.^12^ Thus, it is important that patients’ knowledge of acupuncture and expectations of efficacy are accurate and realistic.

Given the fundamental role of patients’ knowledge and expectations about treatment in influencing decision-making and determining treatment outcomes, it is important to examine levels of knowledge and misconceptions about acupuncture among patients. However, research in this area is limited; previous studies have typically asked respondents to rate their own levels of knowledge 13, 14 yet these responses are subjective and may not reliably indicate actual knowledge levels.^15^ Other studies have focused on practitioners’ knowledge of selected aspects of acupuncture. For example, Lipman, Dale and MacPherson assessed GPs’ knowledge of acupuncture by asking them to describe the difference between western and traditional Chinese acupuncture, to give the name of an acupuncture organisation, and to state whether they had any personal experience of acupuncture treatment.^16^ Other studies have focused on knowledge of complementary and alternative treatments more generally.^17^, ^18^

If knowledge of treatment affects both patient decision-making and treatment outcomes, it is important to establish what knowledge and misconceptions are commonly held, and thus what information needs to be provided to patients. Therefore this study aimed to comprehensively assess knowledge of acupuncture in a sample of people who had, and had not, previously experienced acupuncture.

## 2 Methods

### 2.1 Design

This online cross-sectional questionnaire study forms part of a larger programme of research examining whether providing accurate information about acupuncture and placebo treatments can improve informed choice about these treatments in people with a history of back pain.^19^

### 2.2 Measures

An online questionnaire was constructed to assess knowledge of acupuncture. A pool of items for possible inclusion in the questionnaire was developed after consulting with acupuncturists and complementary medicine researchers, examining literature outlining the empirical evidence for acupuncture efficacy and patient concerns, and reviewing guidelines for acupuncture information sheets and consent processes. 15 items were selected for the study questionnaire to ensure breadth of coverage, including items assessing knowledge of effects, adverse effects, theoretical frameworks, regulation, and access. The draft questionnaire was piloted with 10 members of the public with a history of back pain, recruited through the University of Southampton and support group websites for back pain, and feedback obtained. Minor amendments were made to the wording of the questionnaire items. The final questionnaire comprised 15 true/false items to assess knowledge about: common concerns about acupuncture (6 items); the efficacy of acupuncture for pain (2 items); accessibility of acupuncture (3 items); government legislation of acupuncture (1 item); the forms of acupuncture practiced in the UK (1 item); and, methods of acupuncture administration (2 items).

Additional items assessed demographic characteristics; back pain; and experience of/sources of knowledge about acupuncture. Back pain was assessed with the validated Chronic Pain Grade Questionnaire which asks participants to rate on an 11-point scale current pain intensity and average pain intensity, worst pain intensity, and pain interference with daily activities, recreational or social activities, and work (over the past 6 months).^20^

### 2.3 Participants

Acupuncture-experienced, and acupuncture-naïve people with a history of back pain were recruited for this study. Back pain was selected as an inclusion criterion because there is strong evidence to support acupuncture’s efficacy in treating the condition,^2^ and back pain is commonly treated by acupuncturists in the UK.^1^

### 2.4 Procedure

The study was approved by the Psychology Ethics Committee at the University of Southampton, UK.

Fifteen universities in the UK circulated recruitment emails to students and staff. The study was advertised on social media sites pertaining to back pain, and also emailed to local businesses within the geographical region of Southampton, UK. Recruitment emails asked for people who had ‘had back pain’ to take part in a ‘short (10 min) online quiz’ about alternative treatments for back pain.

People who followed the link to the online questionnaire were shown an information page, including details of the study and agreement to consent to participate in the study. Participants then completed the questionnaire.

Data were imported into SPSS version 22 and summarised using descriptive statistics. *t*-Tests, ANOVA tests, and Spearman’s correlations were conducted to assess whether knowledge of acupuncture was related to previous experience of receiving acupuncture, having read or heard about acupuncture, sociodemographic characteristics (gender, age, ethnicity, highest qualification), and level of back pain and impact on daily living.

## 3 Results

### 3.1 Participant characteristics

226 people took part between July and October 2014. Data were excluded from 24 individuals who failed to complete any of the acupuncture knowledge items, leaving 202 participants. Nine participants failed to specify their age or gender. Of the remaining 193 participants 129 were female (66.8%) and 64 male (33.2%), with an age range from 18 to 74 years (M = 35; *SD* = 14.06). All participants reported having previously experienced back pain: 87.6% (n = 177) in the past six months and 44.1% (n = 89) currently. The intensity of back pain was typically mild to moderate and participants reported interference with daily activities and, to a lesser degree, recreational and work activities. Table 1 presents additional information about participant characteristics.

### 3.2 Acupuncture experience

While most of the sample (77.2%, n = 156) had not previously had acupuncture treatment, 69.8% (n = 141) did report having previously read or heard information about acupuncture. The most frequently accessed sources of information about acupuncture were the Internet (n = 47), family and friends (n = 43), health professionals (including acupuncturists) (n = 42), and other media sources (television/newspapers, magazines) (n = 27).

### 3.3 Acupuncture knowledge

Table 2 summarises responses to the acupuncture knowledge items. Participants answered between 4 and 15 out of the 15 knowledge items correctly (M = 11.03; SD = 2.64). In total, 73.5% of answers were correct. Participants had comparatively high levels of knowledge about common concerns, acupuncture efficacy, and types of acupuncture; these items were answered correctly by at least 80% of all participants. In particular, 95.5% of participants knew that acupuncture can help to relieve pain and 90.5% knew that acupuncture can release natural pain-killing chemicals in the brain. Participants had lower levels of knowledge about accessibility, Government legislation, and methods of administration. Only 56.7% knew that acupuncturists sometimes pass small electrical currents through the needles, and just 43.1% knew that acupuncture can be provided in a multi-bed setting. Importantly, just 66.2% knew that acupuncture could be available on the UK National Health Service (NHS), and only 50.2% of participants knew that acupuncturists are not subject to statutory regulation in the UK.

Those with previous experience of receiving acupuncture were more knowledgeable about the modality than those who had never received treatment with acupuncture (M = 11.8 (SD = 2.16) and M = 10.8 (SD = 1.97), respectively; p < 0.01). As shown in Table 2, significantly more acupuncture-experienced participants correctly answered questions about: likelihood of bruising from needling, thickness of acupuncture needles, availability on the NHS, financial cost, and electro-acupuncture. Although not statistically significant, the mean number of correct items for those who reported previously reading or hearing about acupuncture was marginally higher than those who had not read or heard about acupuncture (M = 11.1 (SD = 2.13) and M = 10.9 (SD = 1.84), respectively).

There were just two differences in knowledge by sociodemographic and clinical characteristics. Participants who identified their ethnicity as white British were likely to have a better knowledge of acupuncture compared with other ethnicities combined (M = 11.29 (SD = 2.01) and 10.54 (SD = 2.03), respectively; p < 0.05). Average pain level over the previous 6 months was negatively correlated with knowledge score: participants who reported less intense pain during the previous 6 months had a greater knowledge of acupuncture (r_s_ = −0.158; p < 0.05).

## 4 Discussion

Acupuncture is extensively practiced within the UK, with a burgeoning evidence base on effectiveness and safety.^1^, ^2^, ^3^ Patient knowledge regarding acupuncture is known to affect both patient decision-making and treatment outcomes, and the provision of high quality patient information is a tenet of UK health policy.^8^, ^9^, ^10^, ^11^, ^12^ Despite the importance of patient knowledge there is a dearth of research evaluating patient knowledge of the modality, with previous research typically concentrating on subjective assessments of knowledge and/or exploring knowledge of complementary therapies generally.^13^, ^14^, ^15^, ^17^, ^18^ To the authors’ knowledge the present study is the first to objectively assess knowledge amongst those who have and have not received acupuncture, using a sample of people with experience of back pain, drawn from the community.

The findings from this study suggest that overall knowledge of acupuncture is relatively high amongst a highly educated sample of people with experience of back pain. Unsurprisingly those who had previously received acupuncture were more knowledgeable about it than those who had not. However, this was not a large effect: on average, those who had previously received acupuncture answered only one additional question correctly compared to those who had never received acupuncture. Perhaps of greater note is the finding that there was no significant difference in knowledge scores between those who identified as having read or heard about acupuncture compared to those who had not. It is possible that provision of acupuncture within the UK (with an estimated 3.8 million acupuncture treatments delivered in 2009^1^), has led to knowledge of the modality pervading public consciousness, even if people are unaware of having specifically read or heard about the treatment; however, additional research is needed to explore this area further.

Previous research has found that the information presented to patients by acupuncturists contains inaccuracies.^5^ Despite this many participants in this survey appeared knowledgeable about common concerns, types of acupuncture, and acupuncture efficacy. Moreover, knowledge of acupuncture’s efficacy for painful conditions was accurate amongst the overwhelming majority of participants; these results are encouraging given that holding high expectations of treatment success has been found to enhance treatment effects.^10^, ^11^, ^12^

Despite the relatively high levels of acupuncture knowledge found amongst both acupuncture-experienced and naïve participants, key weaknesses in knowledge were identified. Only 66.2% knew that acupuncture could be available on the NHS, while just 50.2% of participants knew that acupuncture was not subject to statutory regulation in the UK. UK NICE clinical guidelines currently recommend acupuncture for low back pain, tension-type headache, and migraine, with all conditions being available to treat with acupuncture within the NHS.^3^ In addition a recent survey revealed that a range of additional chronic pain conditions are frequently treated with acupuncture within the NHS.^1^ Participants’ lack of knowledge of the availability of acupuncture for specific conditions within the NHS may have implications for the utilisation of these services. Likewise, although the Department of Health published proposals for the statutory regulation of the profession in 2004^21^ at the time of writing this has yet to be implemented. As a consequence it is currently permissible for anyone to use the title of acupuncturist in the UK subject only to minor legislation. The fact that so many participants in our survey did not know that acupuncture is not subject to statutory regulation in the UK could have serious implications for the profession and the UK public at large. Patients seeking treatment from an acupuncturist may believe that the practitioner is required by law to have completed a certain level of training, or have an accepted level of competency. They may also assume that acupuncturists who fail to maintain acceptable levels of competency could lose their right to use the title. Neither is presently the case, and greater effort needs to be made by both UK acupuncture professional associations, individual acupuncturists, as well as the UK Government, to ensure that patients are aware of the current lack of statutory regulation within the UK and the implications of this.

The present study is the first to objectively assess knowledge of acupuncture amongst a sample of acupuncture-experienced and naïve people with a history of back pain. Overall knowledge of the modality was relatively high. However, key gaps in knowledge were identified, particularly with regards to the provision of acupuncture in the NHS, and the statutory regulation of the profession.

The study contains some limitations which may have impacted on findings. Participants were more highly educated than the UK population in general, with almost 50% possessing a postgraduate qualification. However, it should be noted that level of education was not related to knowledge of acupuncture in the present study. Participants also had experience of back pain, a common chronic condition for which patients frequently find difficulty in achieving satisfactory relief from conventional medications; people with back pain are known to be amongst the most frequent users of complementary therapies in the UK.^22^, ^23^ It may be that people with a history of back pain have a greater knowledge of complementary therapies which might improve their condition than the general public. Thus, this sample may have greater knowledge of acupuncture (even if they do not recall specifically having read or heard about it), than the UK population at large. Future studies could assess the relationship between clinical condition and knowledge of acupuncture, as well as assessing knowledge of acupuncture in other countries, particularly those within the European Union.

## 5 Conclusions

In this study, a high proportion of people with a history of back pain correctly answered questions about the effectiveness and adverse effects of acupuncture. Important knowledge gaps which need to be addressed include lack of awareness of NHS acupuncture services and the regulatory status of acupuncture.

## Competing interests

The authors declare no competing interests.

## Funding

The study was funded by a grant from Arthritis Research UK (grant reference 20113).

## Figures and Tables

**Table 1:** Participant characteristics.


Characteristic	Number (n)	Percent (%)
Gender^a^		
Female	129	66.8
Male	64	33.2

Ethnic origin		
White British	132	65.3
Other White background	38	18.8
Asian or Asian British	9	4.5
Chinese	7	3.5
Other	14	7.0
Preferred not to state ethnicity	2	1.0

Occupation		
Student	63	31.2
Academic	24	11.9
Administrator/secretary	24	11.9
Researcher	19	9.4
Postgraduate student	18	8.9
Other non-office based work	12	5.9
Teaching	12	5.9
Healthcare professional	8	4.0
Technician or IT worker	7	3.5
Currently not working	7	3.5
Other office based work	7	3.5
Unspecified	1	0.5

Highest level of education		
Secondary school	10	5
Some college	29	14.4
Bachelor's degree	47	23.3
Master's degree	55	27.2
Doctoral degree	44	21.8
Other	17	8.4

Pain^b^, ^c^	Mean	Standard Deviation
Intensity (present)	2.07	2.19
Intensity (average over past 6 months)	4.23	1.93
Intensity (worst over past 6 months)	6.39	2.14
Interference in daily activities	3.83	2.58
Interference in recreational activities	3.07	2.70
Interference in work activities	2.92	2.62

a Nine participants failed to specify their gender.

b Items answered on a 0–10 scale where 10 indicates highest levels of pain intensity/interference.

c Twenty nine participants failed to answer questions relating to their back pain.

**Table 2:** Participants’ knowledge about acupuncture.

Questionnaire Item	Correct answer	Total% correct (n)	% correct acupuncture group (n)	% correct acupuncture naïve group (n)
Common concerns about acupuncture				
Serious side effects of acupuncture are extremely rare.	True	89.1 (201)	88.4 (43)	89.7 (156)
Some acupuncture needles are as thick as a human hair.	True	87.1 (201)	83.7 (43)	87.8 (156)
In 80% of treatments patients experience bruising.	False	83.0 (200)	93.0 (43)	80.0 (155)*
Acupuncture needles are as thick as the needles used for an injection.	False	88.1 (201)	100.0 (44)	84.5 (155)**
Disposable needles are never used in acupuncture.	False	87.1 (201)	86.4 (44)	87.2 (156)
Some people feel dizzy after their first acupuncture treatment.	True	82.8 (198)	86 (43)	81.7 (156)

Accessibility				
Acupuncturists are not allowed to give acupuncture in multi-bed settings.	False	43.1 (197)	52.4 (42)	40.5 (153)
Acupuncture is never available on the NHS.	False	66.2 (198)	83.7 (43)	61.4 (153)**
An acupuncture session typically costs around £80 for an hour	False	46.0 (200)	69.8 (43)	38.7 (155)**

Efficacy				
Acupuncture can help to relieve pain.	True	95.5 (202)	97.7 (44)	94.9 (156)
Acupuncture can release natural pain-killing chemicals in our brains.	True	90.5 (201)	95.3 (43)	89.1 (156)

Types of acupuncture				
The two main types of acupuncture in the UK are ‘Traditional’ and ‘Western’ acupuncture.	True	80.2 (197)	80.0 (40)	80.0 (155)

Government legislation				
The UK government does not regulate acupuncture.	True	50.2 (201)	55.8 (43)	48.1 (156)

Methods of administration				
Acupuncture needles are never inserted in the skin at meridian points.	False	69.3 (194)	82.1 (39)	69.9 (153)
Sometimes acupuncturists pass small electric currents through the needles.	True	56.7 (201)	70.5 (44)	52.9 (155)*

* p < 0.05.

** p < 0.01 (Pearson’s χ^2^).
